# Supplementary material for: AC-73 and Syrosingopine Inhibit SARS-CoV-2 Entry into Megakaryocytes by Targeting CD147 and MCT4
Source: Viruses. 2024 Jan 4;16(1):82. doi: 10.3390/v16010082 (PMC10818282; doi:10.3390/v16010082)
Supplement: Supplementary file 1 [file viruses-16-00082-s001.zip › viruses-2762491-supplementary.pdf]

Supplementary Figures

Supplementary Figure S1

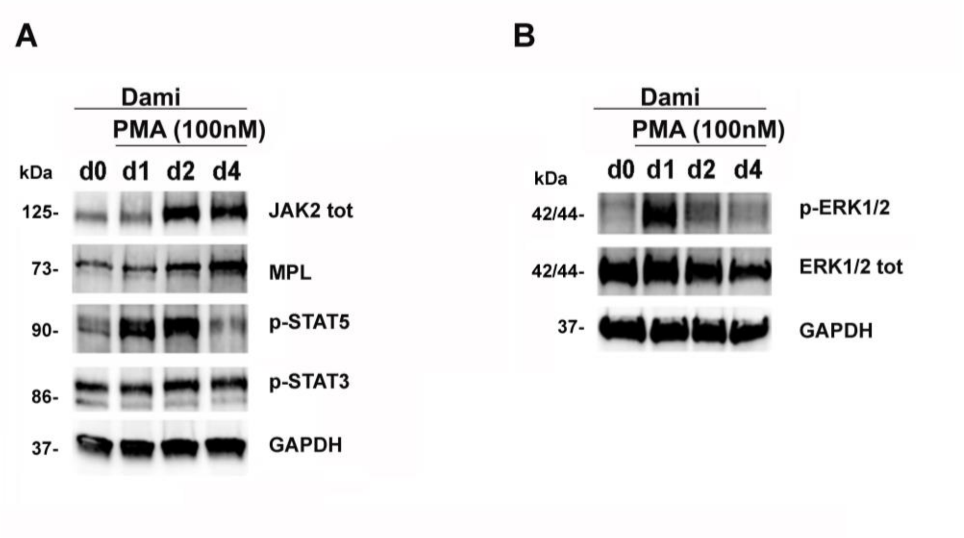

Legend Supplementary Figure S1. PMA (100 nM) treatment of Dami cells, which progressively increases JAK2 and MPL protein level and activates STAT5, STAT3 and ERK1/2 phosphorylation, promotes cell-cycle arrest and MK differentiation of these cells. (**A, B**) Western blot analysis of JAK2 and MPL protein expression and phosphorylation of STAT5 (p-STAT 5), STAT3 (p-STAT 3) and ERK1/2 (p-ERK1/2) during 100 nM TPA treatment of Dami cells; GAPDH is shown as an internal control. Molecular weight (kDa) is indicated. One representative experiment out of three is shown.

Supplementary Figure S2

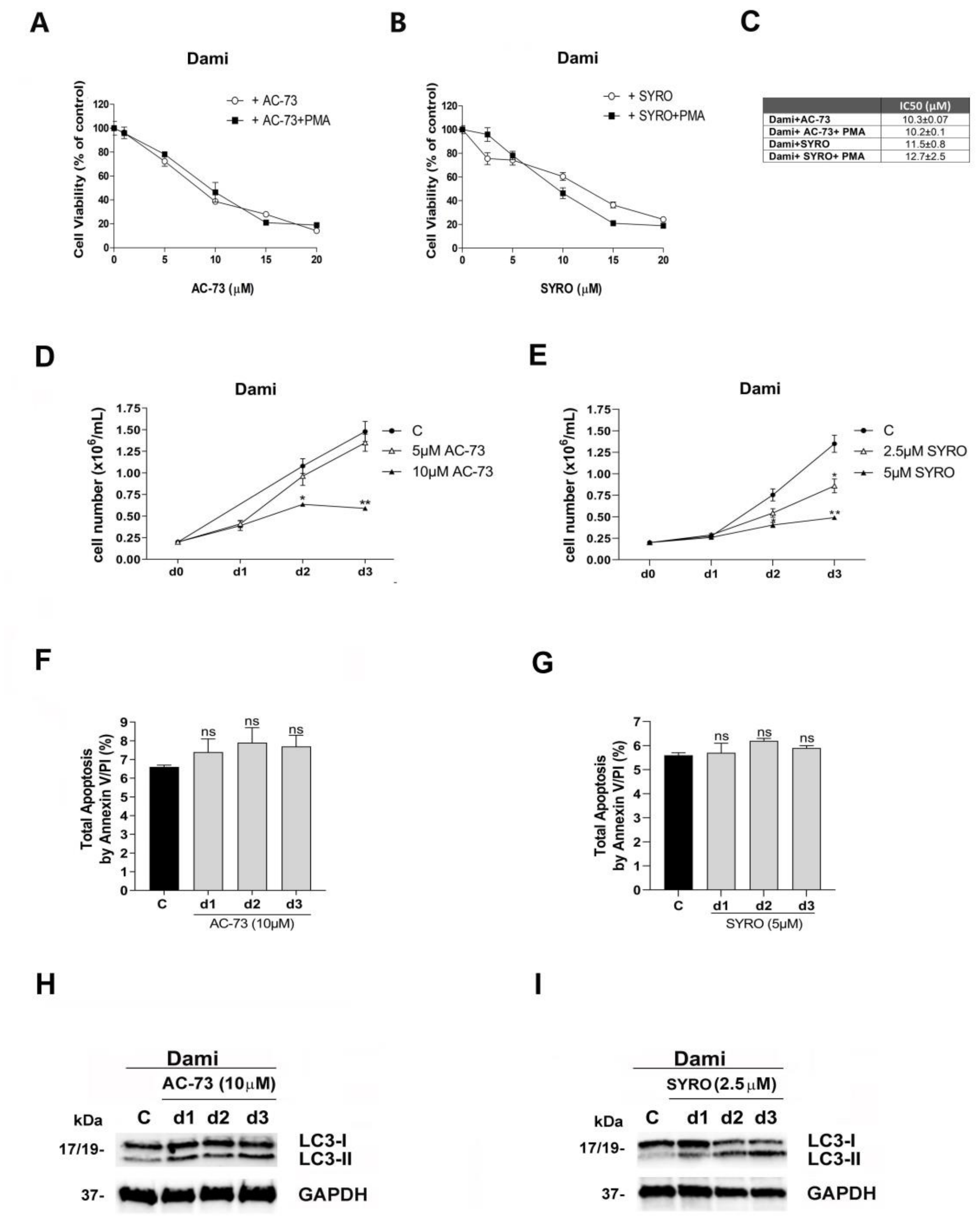

	IC50 (μM)
Dami+AC-73	10.3±0.07
Dami+ AC-73+ PMA	10.2±0.1
Dami+SYRO	11.5±0.8
Dami+ SYRO+ PMA	12.7±2.5

Legend Supplementary Figure S2. Analysis of the effects of AC-73 and SYRO used at different concentrations, on the viability of Dami and PMA-treated Dami cells for IC50 evaluation, on Dami cell growth, apoptosis and autophagy. (**A**) Cell viability assays performed on AC-73 treated Dami cells (AC-73) and AC-73 PMA-Dami cells (AC-73+PMA), as compared to untreated control cells

(0 μM). (**B**) Cell viability assays performed on SYRO treated Dami cells (SYRO) and SYRO PMA-Dami cells (SYRO+PMA), as compared to untreated control cells (0 μM). (**C**) IC50 (μM), the concentration of AC-73 and SYRO whose exhibit 50% cell viability in Dami cells treated with AC-73 (Dami+AC-73) or SYRO (Dami+SYRO) and in PMA-Dami cells treated with AC-73 (Dami+AC-73+PMA) or SYRO (Dami+SYRO+PMA), are reported. (**D, E**) Time and dose response analysis on Dami cell growth indicates that AC-73 (10 μM) and SYRO (2.5 and 5.0 μM) significantly inhibit Dami cell growth, as compared to AC-73 (5 μM)-treated Dami cells (**D**) and to untreated (C) Dami cells (**D** and **E**). (**F**, **G**) No significant apoptosis is found by using AC-73 (10 μM) (**F**) or SYRO (5.0 μM) (**G**), as compared to untreated (C) Dami cells. (**H, I**) AC-73 (10 μM) and SYRO (2.5 μM) induce autophagy in AC-73-treated Dami cells (**H**) and SYRO-treated Dami cells (**I**), as compared to untreated (C) Dami cells, as shown by western blot analysis of the autophagy-related protein LC3-II increased during AC-73 and SYRO treatment (from day 1 to day 3) of Dami cells (**H** and **I**). (**A-G**) Mean ± SEM of three independent experiments is shown. * $p<0.05$; ** $p<0.01$; ns is for not significant. (H, I) One representative experiment out of three is shown; GAPDH is shown as an internal control. Molecular weights are indicated (kDa).

Supplementary Figure S3

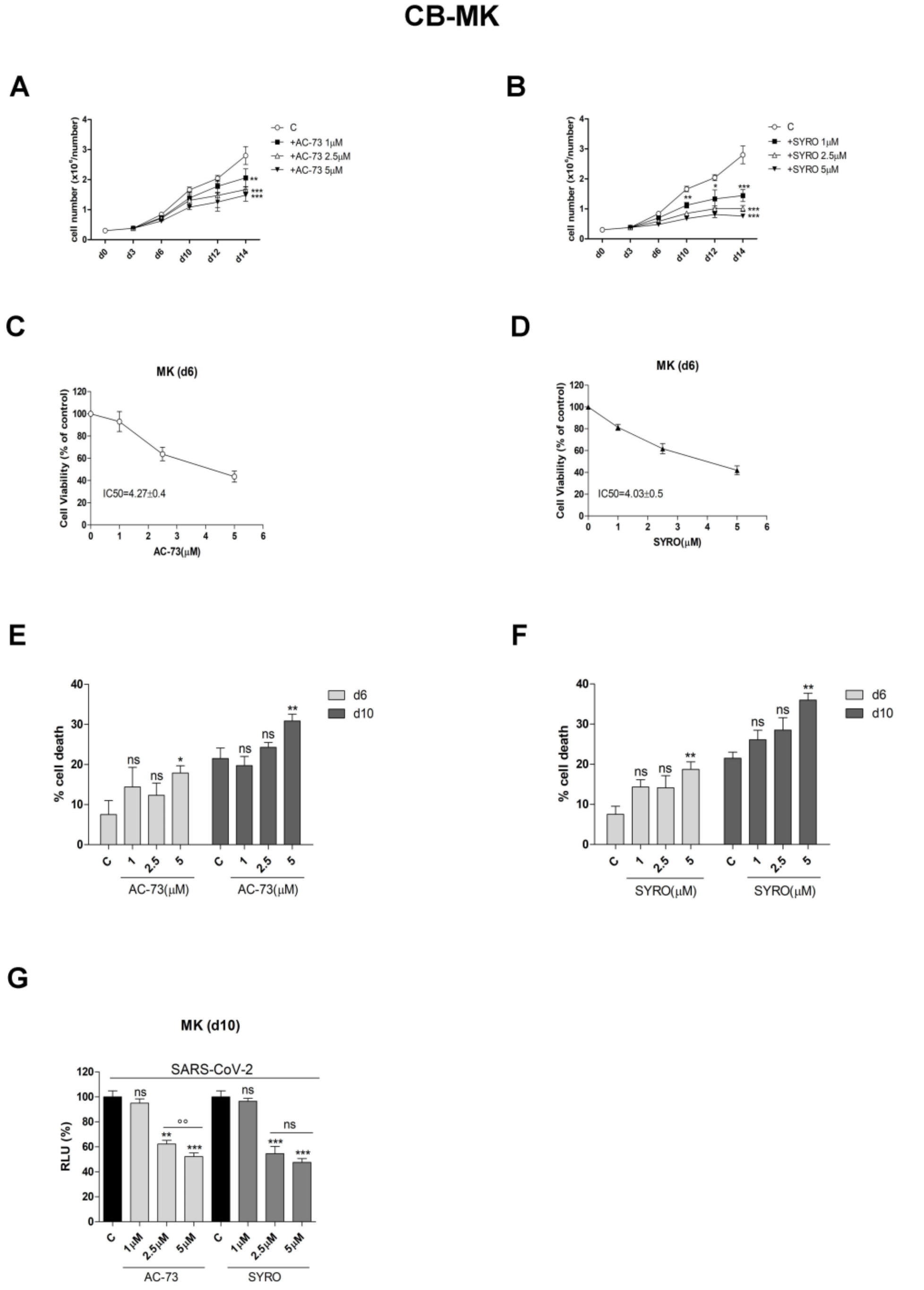

Legend Supplementary Figure S3. Analysis of the effects of AC-73 and SYRO used at different concentrations, on the proliferation, viability and apoptosis during megakaryocytic differentiation of CD34+ hematopoietic progenitor cells (HPCs) purified from human cord blood. (**A, B**) Both AC-73 and SYRO inhibit the proliferation of MK-differentiating HPCs in a dose-dependent manner, as shown by dose response analysis performed by using 1, 2.5 or 5 µM AC-73 (A) and SYRO (B). (**C,**

D) Cell viability assays were performed at day 6 of MK cultures, treated with AC-73 (**C**) and SYRO (**B**), as compared to untreated day 6 MK of control (0 µM) and IC50 (µM), the concentration of AC-73 and SYRO whose exhibit 50% cell viability, evaluated. (**E, F**) A significant apoptosis (% of cell death) is found either at day 6 or day 10 of MK cultures treated with 5 µM AC-73 (**E**) or SYRO (**F**). No significant apoptosis was detected with AC-73 and SYRO used at lower dosage (1 or 2,5 µM) than 5.0 µM in AC-73- or SYRO- treated MKs at day 6 and day 10. (**G**) Luciferase activity detected at day 10 of MK cultures treated with 2.5 or 5 µM AC-73 and SYRO, incubated 2 days with SARS-CoV-2 pseudovirus particles, is significantly lower than the luciferase activity detected in day 10 MK cultures treated with 1 µM AC-73 and SYRO and in day 10 untreated (C) MK (RLU C: 100%). (**A-G**) Mean ± SEM of three independent experiments is shown. *$p<0.05$; **$p<0.01$; ***$p<0.001$. ns is for not significant.
